# Study on morphology optimization of open-pit mine slope based on section and spatial morphology effect

**DOI:** 10.1038/s41598-024-53227-5

**Published:** 2024-01-31

**Authors:** Lan Jia, Jiaqi Wang, Xueting Cai, Linhao Fang, Yonghai Ma, Zhixin Wang

**Affiliations:** 1 College of Mining, Liaoning Technical University, Fuxin, 123000 Liaoning China; 2 Sino Sindh Resources Pvt Ltd, Mithi, 69230 Sindh Pakistan

**Keywords:** Engineering, Mineralogy

## Abstract

Reasonable design of the slopes of mining field is an important prerequisite for optimizing open-pit mine boundary, ensuring safe production and improving economic benefits of open-pit mines. This study took the open-pit coal mine coal mine in Block I of Thar coal field in Pakistan as the research background, based on the section morphology effect of slope stability and the occurrence characteristics of coal seam and weak layer, the section morphology of slope was optimized step by step from two-dimensional angle by using the rigid body limit equilibrium method. The three-dimensional numerical simulation method is used to analyze the three-dimensional spatial effect of slope stability and reveal the influence of the spatial geometry of the slope stability. The spatial morphology of the slope was optimized and the evolutionary mechanism of slope instability was revealed. By comparing the optimized slope morphology with the preliminary design and the slope morphology of the straight slope with equal flat plate, the economic benefits were further analyzed. The results showed that under the premise of meeting the safety reserve coefficient, compared with the preliminary design and the design of equal plate. The slope angle of the optimized section morphology was increased by 1° and 3° respectively, and the coal resource recovery was increased by 332 m^2^ and 1790 m^2^ respectively, and the economic benefits were $ 18,065,859 and $ 54,408,251 respectively. And the slope angle of the optimized spatial morphology was increased by 3° and 5° respectively, and the coal resource recovery was increased by 1188 m^2^ and 2726 m^2^ respectively, and the economic benefits were $ 72,082,808 and $ 108,417,368 respectively.

## Introduction

The problem of spatial morphology of open pit mine slopes is both a safety problem and an economic problem. How to scientifically evaluate the stability of multi-weak layer soft rock slopes with respect to the unique engineering and geological characteristics of different open-pit mines, and the significant difference in slope morphology on the slope stability, were worthy of concerns in the field of safety engineering of open-pit mine slopes. And it was of great significance to put forward reasonable slope morphology optimization methods based on slope morphology effects to ensure that open-pit mines achieve efficient and safe production.

Some scholars at inland and abroad had carried out a series of studies on slope morphology optimization design. Li et al.^1^ considered the differences in stratigraphic lithology in the vertical direction of stratified slopes in open-pit mines, the service time in the process of engineering development, the required degree of stability, as well as the three-dimensional effect of the stability of deeper slopes, and used a combination of the two-dimensional limit equilibrium method and three-dimensional numerical simulation to optimize the morphology of slopes of fault-containing and paralleling weakly dipping groups of slopes in open-pit mines with subdivided segments. Xu et al.^2^ by analyzed the geological conditions of slope engineering and the characteristics of stratum occurrence, the slope stability of open-pit coal mine under complex geological conditions was studied, and proposed a level-by-level optimization method of slope morphology from top to bottom with each weak layer as the slip surface. Wang et al.^3–5^ conducted numerical simulation studies on the stability of inclined soft rock slope in the open-pit mine under different spatial morphology. It was concluded that the slope stability decreases with the increase of tracking distance, and the effect was remarkable in field implementation. And meanwhile, they studied the influence of the slope section morphology on the stability of the slopes, and put forward the concept that the morphology effect produced by a reasonable convex slope is beneficial for the stability of the slope, which has a significant advantage in the reduction of the stripping costs.

Li^6^ and Tian^7^ considered the effect of the rotational radius of the slope and the lateral earth pressure of soil strip on the slope stability, and established a three-dimensional stability analysis system of convex and concave slopes based on Swedish arc method and Bishop method. Lu et al.^8^ studied the limit stable angle of convex slope and flat slope through the bottom lifting device. And the theoretical and experimental studied on the effect of slope form on slope stability. It was concluded that the ultimate stability angle of concave slope was larger than that of flat slope, while that of convex slope was smaller than that of flat slope. At the same time, the stress difference between convex slope, flat slope and concave slope was analyzed in theory, the phenomenon that concave slope was more stable than convex slope was explained.

In foreign countries, BAKER^9,10^ and Farzaneh et al.^11^ used variational limit equilibrium method and limit analysis method respectively. It was concluded that the stability of circular convex slope decreased with the increase of R/H (where R was the Curvature radius of slope and H was the height of slope), and tend to the calculation result of plane strain. Xing^12^ discussed the failure mode of concave slope and studied the three-dimensional stability analysis method. He believed that its size was related to the curvature ratio. Rassam and Williams^13^ studied the relationship between the morphology of the mine waste rock pile and the stability. It was concluded that the limit stable angle of concave slope was at least 2° larger than that of flat slope, and the limit stable angle of convex slope was 0.5° smaller than that of flat slope.

There are a lot of research results on the slope stability of open-pit coal mine. However the problem of soft rock slope stability of open-pit mine with significant time effect and spatial effect has not been solved. The key is that there is still a lack of necessary scientific methods for the optimization design of slope spatial morphology, so that it can just meet the stability requirements of a certain service life and achieve the coordinated control of mining procedure and slope spatial morphology. In summary, this study took the open-pit coal mine in Block I of Thar coal field in Pakistan as the research background, and adopts the rigid-body limit equilibrium method combined with numerical simulation, analyzed the slope section morphology effect and spatial morphology effect, optimized the design of to-boundary slope morphology. Finally, it compared the economic benefits generated by different slope morphologies, which fully highlight the potential economic benefits of the section morphology effect and spatial morphology effect. This can provide guidance and reference for similar soft rock open-pit coal mines. Especially open-pit coal mine slope engineering design and treatment in Pakistan. At the same time, it will enrich the soft rock slope stability analysis and open-pit mine design theory and method, so its scientific significance is great.

## Geological background of slope engineering

**Figure 1:**
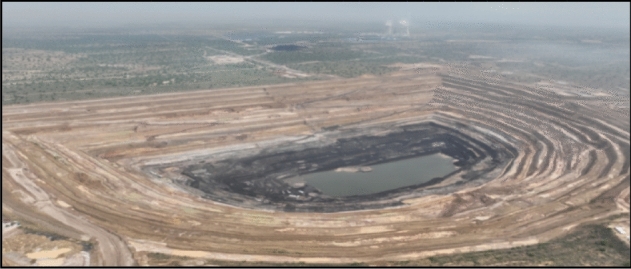
Mining status of open pit mine.

**Figure 2:**
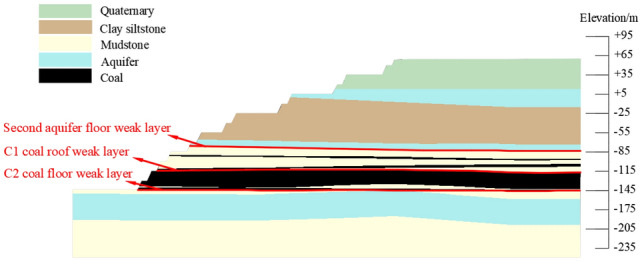
Engineering geological profile of slope.

The designed production capacity of an open-pit coal mine in Thar Coalfield, Pakistan was 7.8 million tons per year. The main rock (soil) layers in the slope can be divided into three stages : Quaternary aeolian sand layer (I), Neogene Pliocene soft rock layer (II), Paleogene Paleocene-Eocene soft rock layer (III). The Tar coalfield in Pakistan is an extremely wide and gradual syncline along the NNE direction. Layers occurrence gentle, the dip angle is generally about 2°. The undulating coal seam basement leads to slight undulation in the lower part of coal measures strata. Three key weak layers are developed in this area, whose lithology is mainly mudstone, carbonaceous mudstone or clayey siltstone. The mudstone is characterized by high content, loose structure and poor stability. But its water absorption and water holding capacity are strong, and it is easy to soften, expand and disintegrate after encountering water. Three aquifers are developed in the area, all of which are porous aquifers. Among them, the Quaternary sand dune aquifer is a phreatic aquifer. While the Neogene Pliocene bottom sandstone aquifer of the coal seam roof and floor and the Paleogene Paleocene-Eocene bottom sandstone aquifer are confined aquifers. The mining status of open pit mine was shown in Fig. 1, and the engineering and geological profile of the slope was shown in Fig. 2.

According to the control theory of rock mass structure, the failure mode of slope mainly depends on the development of joints and fissures. Slope instability mainly depends on the relationship between anti-sliding force and sliding force^14^. The slope of the open-pit coal mine is mainly controlled by the weak layer of the second aquifer floor in the slope, the weak layer of the C1 coal roof, and the weak layer of the C2 coal floor. The potential landslide mode is analyzed as arc sliding or cutting-bedding sliding with the key weak layer as the bottom interface.

According to the provisions of the slope safety coefficient in the ‘Code for design of open pit mine of coal industry’ (GB50197-2015), and considered the importance of the slope and the proven degree of the occurrence conditions, the safety reserve coefficient of the slope slope was 1.3. Determined physical and mechanical parameters of slope and base rock-soil mass based on engineering geological conditions. The physical and mechanical indexes of each layer were shown in Table 1.

**Table 1:** Physical and mechanical indexes of rock-soil mass.

Rock formation	Cohesion *C*/kPa	Internal friction angle *φ*/°	Volumetric weight *γ*/kN m^−3^	Modulus of elasticity *E*/Mpa	Poisson’s ratio *μ*
Quaternary fine sand	10	33	20	20	0.38
Clay siltstone	110	22	21	1700	0.25
First aquifer	71	19	19.56	2750	0.35
Second aquifer	10	31	21	2355	0.27
Third aquifer	5	35	19	2400	0.27
Mudstone	100	18	18	1207	0.35
Coal	180	30	12	2200	0.28
Weak layer	5	18	20	300	0.24

## Optimization of slope section form

### Analysis of the section form effect mechanism

**Figure 3:**
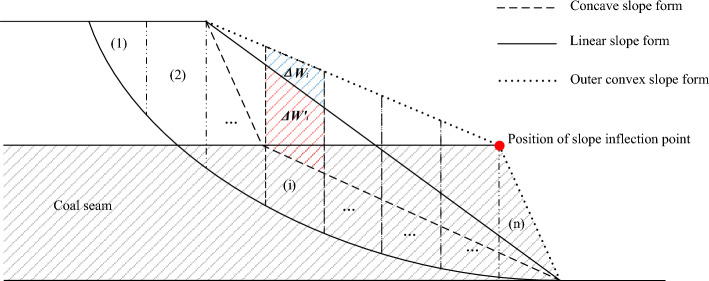
Convex, concave and flat slope morphology.

Slope is formed by engineering geological conditions and artificial excavation in mining area. Its stability is closely related to section morphology and slope parameters. Slope section morphology can be divided into linear slope (straight slope) and non-linear slope, non-linear slope can be divided into convex, concave two kinds of morphology. In the practice of open-pit mining engineering, the difference between nonlinear slope and linear slope in morphology has an essential difference in the influence of slope stability, which is called the section morphology effect of slope. For the convex and concave slopes with equal overall slope angles, Wang et al.^5^ compared and analyzed the slope stability of nonlinear slopes with different degrees of convexity and linear slopes from the perspective of mechanics, and revealed the mechanism of section morphology effect of slopes, and the morphologies of Convex, concave and flat slopes were shown in Fig. 3. The slices of convex, concave slope and the slices of flat straight slope shown in Fig. 3 have the weight difference *△W*_*i*_, *△W′*_*i*_ respectively. Compared with convex slope and linear slope, *△W*_*n*_ gradually increases from top to bottom. This trend will be beneficial to the critical anti-sliding force increment at the bottom of each block on the lower side of the sliding surface. And the inclination angle of bottom slip surface gradually decreases, so that the anti-sliding force of the whole sliding body increases, which is more favorable to the overall stability of the slope. However, comparing concave slope and linear slope, the conclusion is completely opposite. Therefore, section convex slope is more favorable to slope stability than concave slope and straight slope. This method not only reduces stripping and increases resource recovery, but also improves the stability of each slope.

### Section form optimization scheme

**Figure 4:**
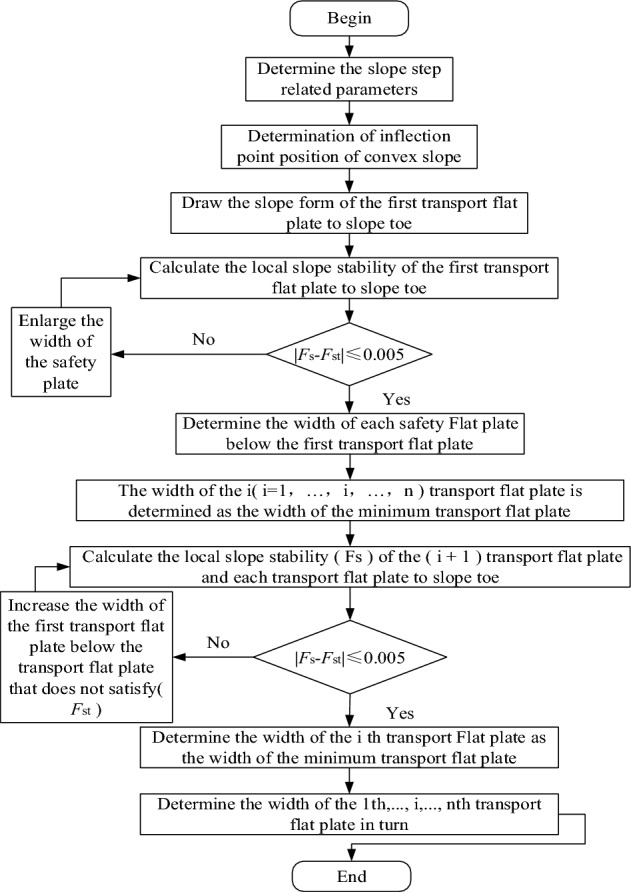
Flow chart of section form optimization.

**Figure 5:**
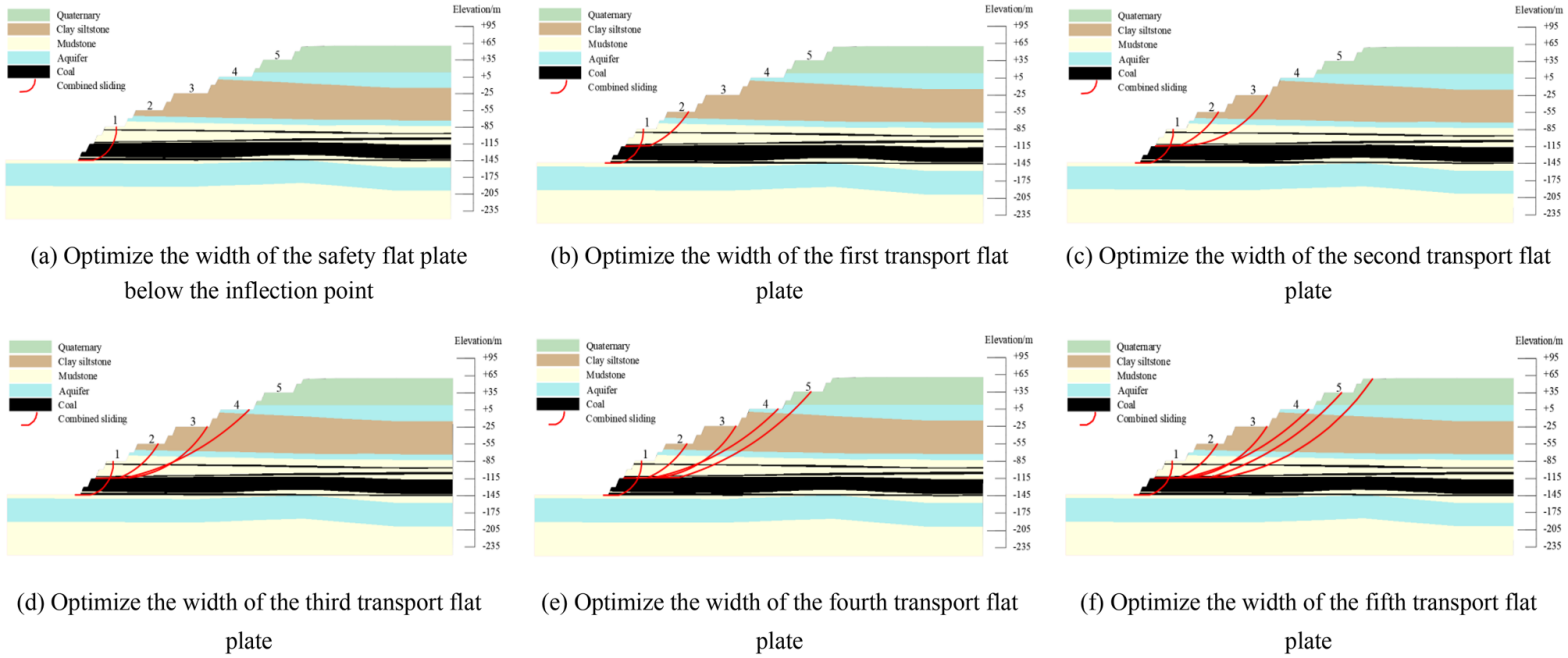
Schematic diagram of the optimization principle of section form (taking the weak layer of the C1 coal roof as the bottom slip surface as an example).

According to the above-mentioned slope section morphology effect mechanism, the boundary slope was designed as an outer convex slope surface, so that the slope becomes a convex slope morphology with the upper ease and the lower steep. Through a large number of trial calculations, it was determined that the most dangerous sliding surface was the cutting-bedding sliding surface with the weak layer of C1 coal roof as the bottom sliding surface. Therefore, in optimization design, only the slope stability calculation results in this sliding mode were considered. The flow chart of the section morphology optimization step was shown in Fig. 4. Taking the south side as an example, the schematic diagram of the section morphology optimization principle was shown in Fig. 5. The optimization steps are explained as follows:

① According to the actual production, determine the height of the steps, the minimum width of the transport flat plate and the width of the safety flat plate, the elevation of each transport flat plate, the slope angle of the steps and the safety reserve coefficient *F*_st_. Accordingly, design a set of composite steps consisting of a safety flat plate and a transport flat plate.
② According to the distribution characteristics of rock strata in the mine, it is drafted that the slope top of the first transport flat plate above the roof of the coal seam is the inflection point of the convex slope, namely – 85 m level.
③ According to the principle of one by one flat plate optimize from slope toe to slope top. First, adjust the width which is the bottom of the pit to the safety flat plate below the first transport plate until the slope at this stage meets the requirements of the safety reserve coefficient *F*_st_, thereby determining the width of each safety flat plate below the first transport flat plate.
④ Follow these steps to optimize from the first transport flat plate. It is drafted that the width of the i (i = 2……n) transport flat plate is determined as the width of the minimum transport flat plate, and the width of the safety flat plate is determined as a fixed value (the width of the minimum safety flat plate). Calculate the local slope stability of the (i + 1) transport flat plate and each transport flat plate below it to slope toe.. If the stability coefficient of the (i + 1) transport flat plate is less than the safety reserve coefficient, it is necessary to increase the width of the i transport flat plate until all local slopes meet the requirements of the safety reserve coefficient. So as to complete the determination of the width of the first to n transport flat plates. If the (i + 1) stability coefficient is greater than the safety reserve coefficient, the width of the i transport flat plate is the width of the minimum transport flat plate until the optimization of the width of all flat plates work is completed.

### Method of slope stability analysis

At present, the most commonly used method for quantitative analysis of slope stability is the rigid body limit equilibrium method. The method is widely used in engineering because of its simple calculation process and reliable results^15–17^. The basic principle of the rigid body limit equilibrium method is to regard the rock-soil mass as a rigid body without considering the deformation problem. And it analysis the relationship between the slip resistance and sliding force to evaluate the slope stability. At present, the most commonly used slope stability analysis methods are Fellenious method^18^, simplified Bishop method^19^, Sarma method^20^, residual thrust method^21,22^. The simplified Bishop method is suitable for circular sliding surface landslide. The residual thrust method is suitable for any sliding surface landslide. Based on the above two analysis methods, a rigid body limit equilibrium analysis software is developed for quantitative analysis of slope stability.

### Results of section morphology optimization design

**Figure 6:**
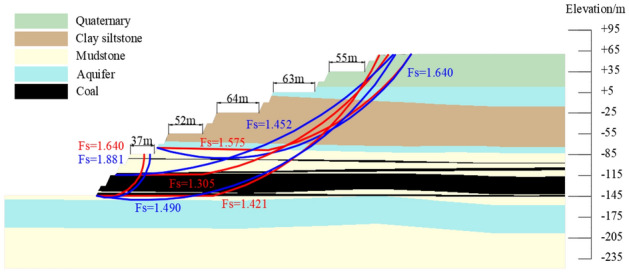
Typical profile slope optimization design results.

According to the above slope morphology optimization scheme, the typical profile of south side was optimized. The section morphology and slope stability calculation results of the optimized profile were shown in Fig. 6. After the optimized design of the south side, the slope stability coefficient was 1.305, and the landslide mode was cutting-bedding sliding with the C1 coal top plate as the bottom interface. The global and local slope stability coefficients all could meet the requirement of safety reserve coefficient 1.30. The final slope angle was 25°, with the first transportation flat pan + 85 flat pan above the roof of the main mining seam as the inflection point, the step side slope angle of the above stage was 23°, the step side slope angle of the following stage was 48°, the step slope angle was 65°, the width of each transport plate was 37 m, 52 m, 64 m, 63 m, 55 m, and the width of the safety plate was 8 m.

## Spatial form optimization of boundary slope

### Spatial form optimization scheme

The stability of open-pit slope is not only affected by engineering geological conditions, it is also closely related to the spatial geometry and parameters of the slope formed by mining engineering activities. Therefore, design and the stability analysis of open-pit slope should be considered together with the mining procedure. The essence of the effect of horizontal mining procedure on slope stability was to reduce the exposure length and bare time of the slope, so as to improve and enhance the stability^23^. This is the use of the double supporting effect of the horizontal mining working side and the inner dump on the slope. This effect is mainly reflected in the deep slopes. As the slope transitions to the shallow part in the vertical direction, the due to the slope exposure length becomes larger and larger, the effect will gradually decrease or even disappear.

According to the results of two-dimensional slope stability analysis, the most dangerous slip surface was an cutting-bedding combined slip surface with the weak layer of C1 coal seam roof as the bottom slip surface. So determined the lowest pressure side height need to reach C1 coal seam roof weak layer level elevation above the first transport flat plate – 85 m level. Because the stability coefficient of the local slope above the second aquifer was much larger than the requirement of the safety reserve coefficient 1.30. And the upper part was mostly rock which did not need further stripping. Therefore, on the basis of the optimization of the two-dimensional slope morphology, the slope angle could be further increased by shortening the width of the flat plate at the levels of – 85 m and – 55 m. This could increase the amount of coal mining. In order to determine the reasonable slope spatial morphology, three-dimensional numerical simulation methods were used to analyze the influence of slope angle and tracking distance on slope stability. Designed three three-dimensional numerical simulation schemes were as shown in Table 2.

**Table 2:** Three-dimensional stability research scheme of boundary slope.

Name of scheme	The width of the flat plate at − 85 m/m	The width of the flat plate at − 55 m/m	Angle/° /°
Scheme 1	37	52	25
Scheme 2	25	52	26
Scheme 3	25	32	27

### Numerical simulation analysis of slope form

1. Model construction

**Figure 7:**
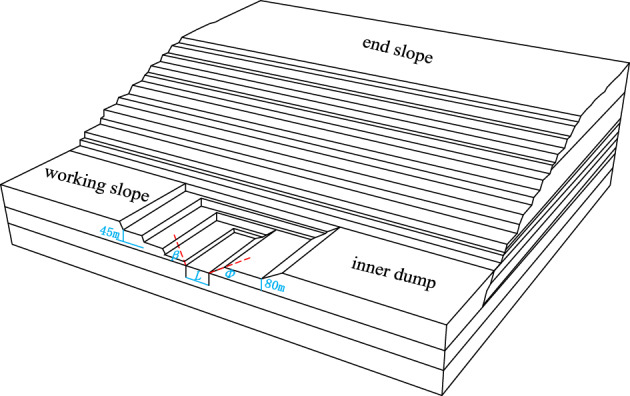
Diagram of three-dimensional numerical simulation model.

A three-dimensional numerical simulation model of slope was established when the slope was pressed to − 85 level respectively. Each model consists of three parts : south side, horizontal mining working side and inner dump. Working slope angle *β* and inner dump slope angle *α* remain unchanged (*β* = 70°, *φ* = 33° as determined by the minimum working flat plate of 45 m for horizontal mining and the minimum working flat plate of 80 m for inner dump). Only changed the tracking distance *L* between the working side and the inner dump, and the angle of the boundary slope. The numerical simulation models under three excavation angles (25°, 26°, 27°) and five tracking distances (50 m, 100 m, 200 m, 300 m, 400 m) were established respectively. In order to avoid the influence of boundary effect on the calculation results, the size of the model should meet the recommendations and requirements of Goodman. In the model, the slope surface of the south side, the slope surface of the horizontal mining working side and the slope surface of the inner dump were all free surfaces. And the surrounding and bottom of the model were constrained by horizontal and vertical displacements respectively. Create a model as shown in Fig. 7.

2. Numerical simulation results analysis

**Figure 8:**
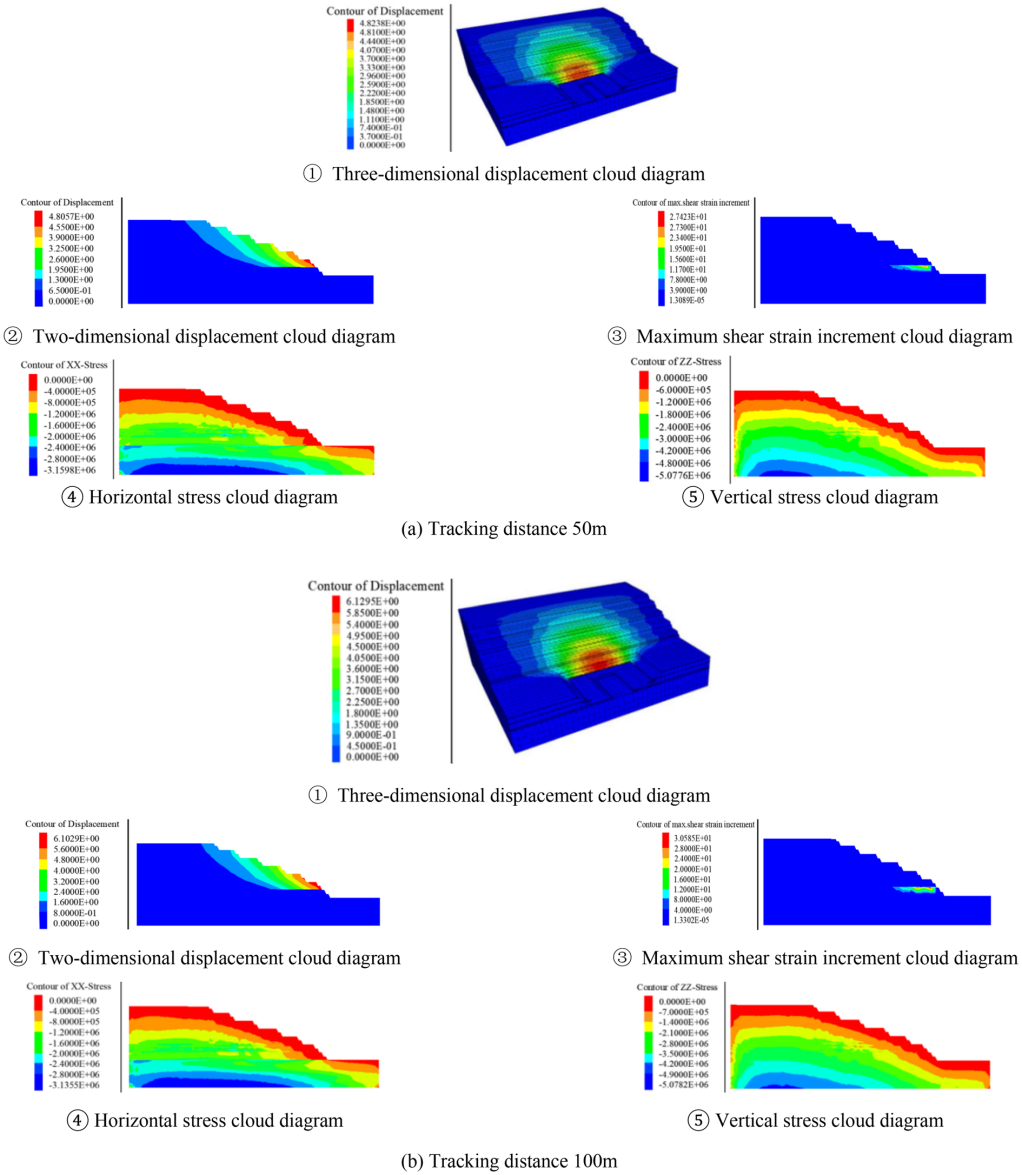

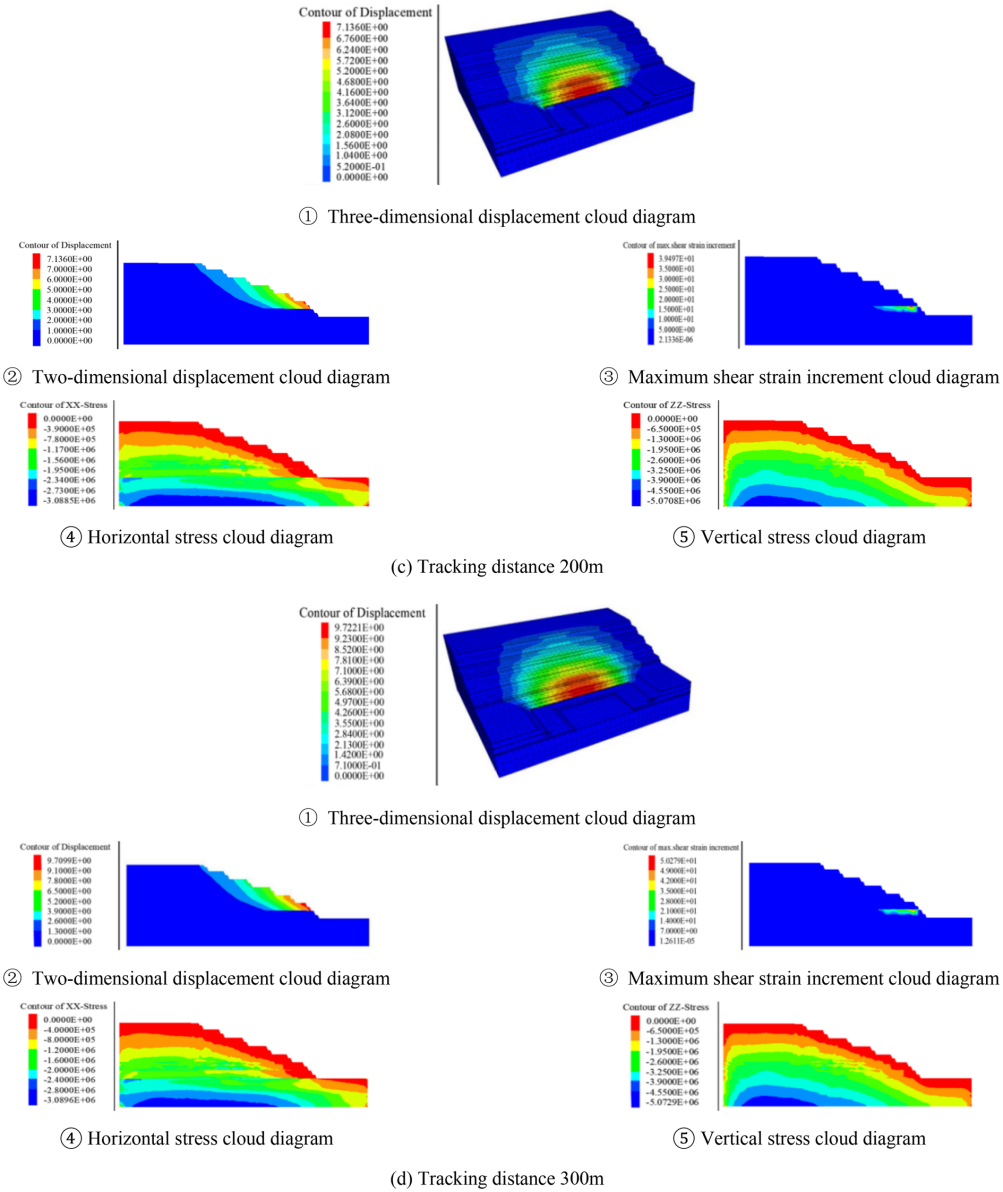

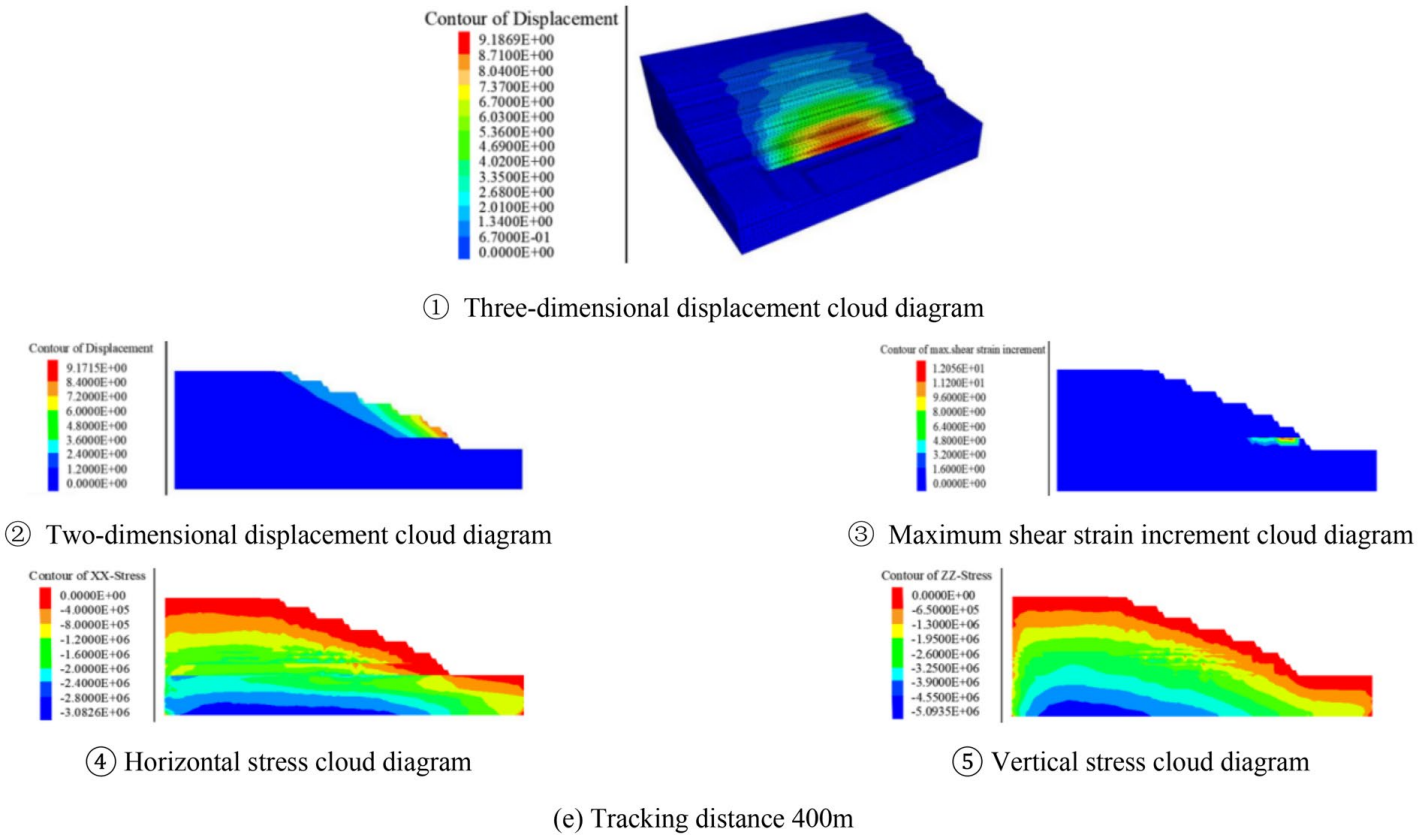
Numerical simulation results when excavating at 25°

Before the open pit mining, the initial ground stress field was generated by gravity, and the initial vertical ground stress was distributed in layers. After generating the initial ground stress field, it was assumed that the excavation formed slopes under the conditions of different excavation angles and tracking distances, and then calculates to equilibrium respectively. Through numerical simulation, the stability coefficient and the deformation damage characteristics at critical instability could be obtained. Taking the section morphology inclination angle of 25° as an example, the calculation results under different tracking distance conditions were shown in Fig. 8.

**Figure 9:**
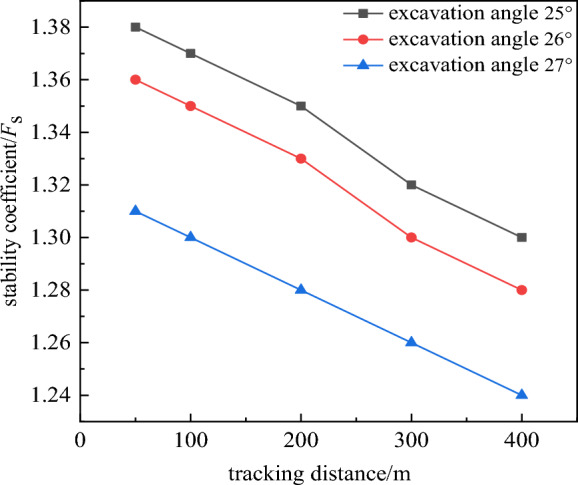
Relationship between tracking distance and stability coefficient.

**Figure 10:**
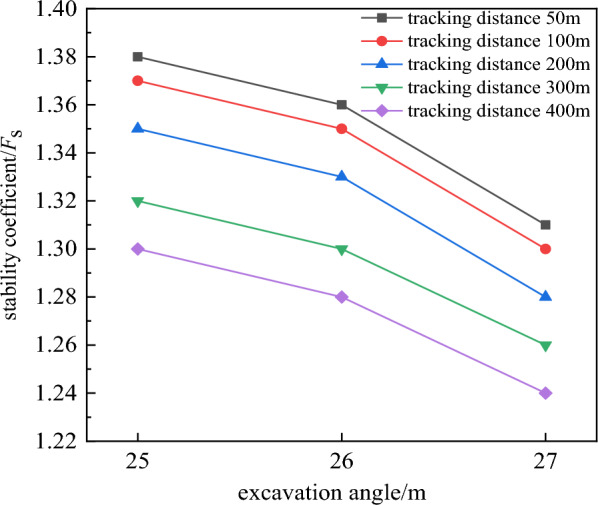
Relationship between excavation angle and slope stability coefficient.

It can be clearly seen from the relationship between the tracking distance and the stability coefficient in Fig. 9 that the tracking distance has a great influence on the deformation and stability of the slope. It can be seen that the slope stability coefficient decreases linearly with the increase of the tracking distance. From the relationship between the excavation angle and the slope stability coefficient in Fig. 10, it can be seen that the near linear law decreases with the increase of the excavation slope angle, and the deformation and deformation range of the rock mass gradually increase. When the tracking distance was less than 100 m, the displacement of the deep rock mass was relatively small due to the double support of the working side and the inner dump. However, no matter how the tracking distance decreased, due to the weak layer of C1 coal roof and the weak layer of the second aquifer floor were far away from the bottom, and the opening distance of the weak layer of C1 coal roof reaches 280 m. The above rock masses were not subjected to the double supporting effect of the working side and the inner dump, so the potential landslide mode of the slope was the cutting-bedding sliding with the C1 coal roof as the bottom interface. This was consistent with the calculation results of the two-dimensional limit equilibrium method, and the position and morphology of the slip surface were also close. This showed the calculation results had good reliability.

### Results of spatial morphology optimization design

**Figure 11:**
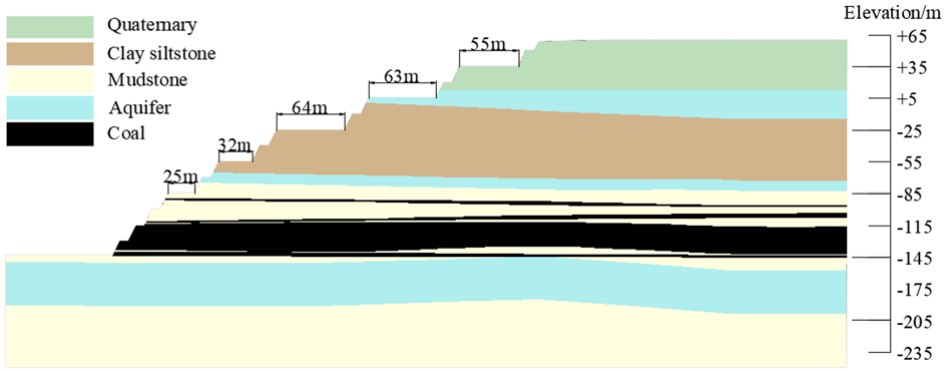
Slope section form after spatial optimization.

According to the numerical simulation results, when the slope angle was 27°, the tracking distance was 50 m and 100 m, the slope stability coefficient was 1.31 and 1.30. It just met the requirement of safety reserve coefficient 1.3. The mastery degree of rock-soil mass physical mechanics index and the influence of aquifer were considered, taking into account the limited working conditions required for mining equipment and the impact of transport distance. The tracking distance was determined to be 50 m, the overall slope angle was determined to be 27°. The width of the transport flat plate was 25 m and the width of the safety flat plate was 8 m. The slope section form was shown in Fig. 11.

## Result and discussion

### Stability analysis of the preliminary design slope

**Figure 12:**
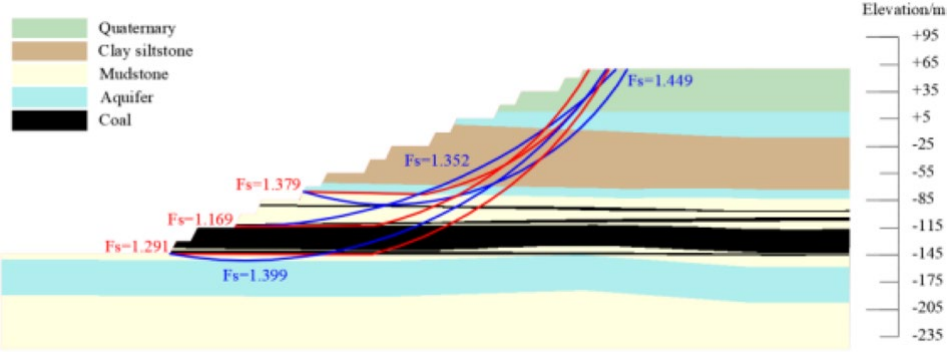
Preliminary design to boundary slope stability calculation results.

The slope stability of the preliminary design scheme for the above typical profile is evaluated based on the Bishop method and the residual thrust method in the rigid body limit equilibrium method. The preliminary design to the boundary slope consists of a safety flat plate and a transportation flat plate to form a group of combined steps, the safety flat plate was 16 m, the transportation flat plate was 40 m, and the final slope angle was 24°. The result of slope stability coefficient calculation was 1.169, and the landslide mode was cutting-bedding sliding with the C1 coal top plate as the bottom interface, which couldn't meet the requirement of safety reserve coefficient 1.30. The result of slope stability analysis was shown in Fig. 12.

### Stability analysis of the straight slope with equal flat plate

**Figure 13:**
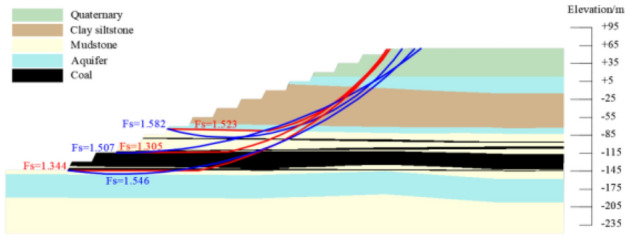
Straight slope with equal flat plate slope stability calculation results.

Designing the equal flat plate scheme for the typical profile described above, based on a safety reserve factor of 1.3. The design slope angle was 22°, the flat plate width was 32 m, the slope stability stability calculations was 1.305, and he landslide mode was cutting-bedding sliding with the C1 coal top plate as the bottom interface. The result of slope stability analysis was shown in Fig. 13.

### Discussion

1. Comparative analysis of slope morphology after section optimization with preliminary design slope and the straight slope with equal flat plate

**Figure 14:**
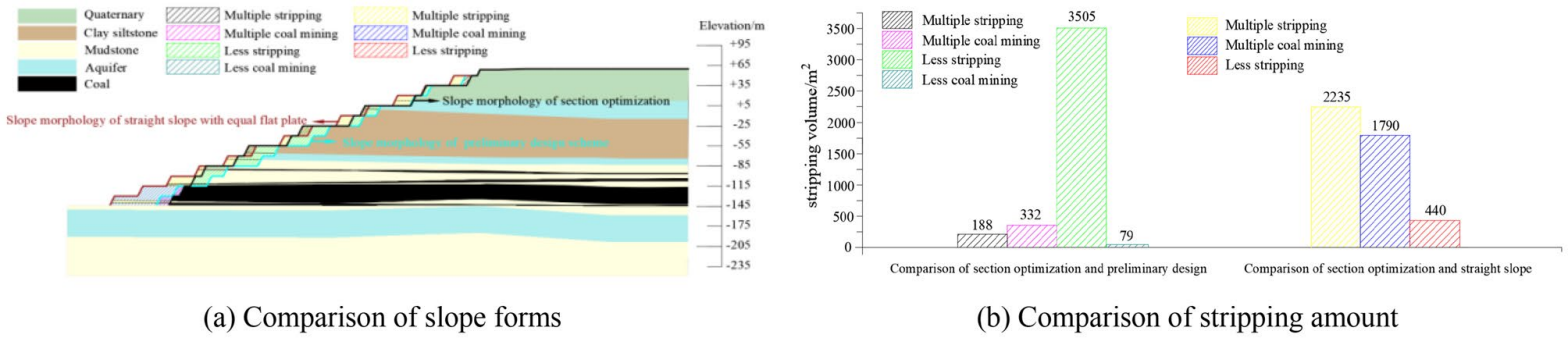
Comparison of the slope morphology and the stripping amount of the slope after section optimization with the preliminary design slope and the straight slope with equal flat plate.

The optimized morphology of the section was compared with the preliminary design slope and the straight slope with equal flat plate. The stripping amount comparison was shown in Fig. 14. The slope angle, stability coefficient and economic benefit were compared as shown in Table 3.

**Table 3:** Comparison of the stability and economic benefits of the slope after section optimization with the preliminary design slope and the straight slope with equal flat plate.

Form of slope	Angle of slope/°	Stability coefficient	Economic benefit/$
Preliminary design	24	1.169	18,065,859
Straight slope	22	1.305	72,082,808
Section optimization	25	1.305	–

Compared with the slope of preliminary design scheme, after section morphology optimized the slope angle was increased by 1°, the stability coefficient was increased by 0.136, the stripping amount of section measurement was increased by 188 m^2^, and the recovery amount of coal resources was increased by 332 m^2^. According to the horizontal length of 1 km to calculate, if the price of per ton of coal was $ 38.35, the cost of stripping was $ 2.2125/m^3^, the cost of coal mining was $ 2.95/t, the average per kilometer could produce benefit of $ 18,065,859 after the morphology optimization. Compared with the straight slope with equal flat plate, after section morphology optimized the slope angle was increased by 3°, the stability coefficient was increased by 0.141, the stripping amount of section measurement was increased by 2235 m^2^, and the recovery amount of coal resources was increased by 1790 m^2^. According to the horizontal length of 1 km to calculate, the average per kilometer could produce benefit of $ 72,082,808 after the morphology optimization. In summary, the section morphology optimization scheme was better than the design scheme of the straight slope with equal flat plate and the preliminary design scheme, and the economic benefit was significant.

2. Comparative analysis of slope morphology after spatial optimization with preliminary design slope and the straight slope with equal flat plate.

**Figure 15:**
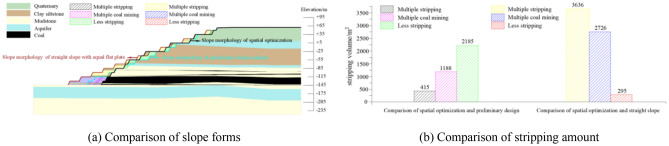
Comparison of the slope morphology and the stripping amount of the slope after spatial optimization with the preliminary design slope and the straight slope with equal flat plate.

The optimized morphology of the spatial was compared with the slope of preliminary design scheme and the straight slope with equal flat plate. The stripping amount comparison was shown in Fig. 15. The slope angle, stability coefficient and economic benefit were compared as shown in Table 4.

**Table 4:** Comparison of the stability and economic benefits of the slope after spatial optimization with the preliminary design slope and the straight slope with equal flat plate.

Form of slope	Angle of slope/°	Stability coefficient	Economic benefit/$
Preliminary design	24	1.169	54,408,251
Straight slope	22	1.305	108,417,368
Spatial optimization	27	1.31	–

Compared with the slope of preliminary design scheme, after spatial morphology optimization the slope angle was increased by 3°, the stability coefficient was increased by 0.141, the stripping amount of measurement was increased by 415 m^2^, and the recovery amount of coal resources was increased by 1188 m^2^. According to the horizontal length of 1 km to calculate, if the price of per ton of coal was $ 38.35, the cost of stripping was $ 2.2125/m^3^, the cost of coal mining was $ 2.95/t, the average per kilometer could produce benefit of $ 54,408,251 after the morphology optimization. Compared with the straight slope with equal flat plate, after spatial morphology optimization the slope angle was increased by 5°, the stability coefficient was increased by 0.005, the stripping amount of measurement was increased by 3636 m^2^, and the recovery amount of coal resources was increased by 2726 m^2^. According to horizontal the length of 1 km to calculate, the average per kilometer could produce benefit of $ 108,417,368 after the morphology optimization. In summary, the spatial morphology optimization scheme was better than the design scheme of the straight slope with equal flat plate and the preliminary design scheme, and the economic benefit was significant.

3. Comparative analysis of slope with optimized section morphology and slope with optimized spatial morphology.

**Figure 16:**
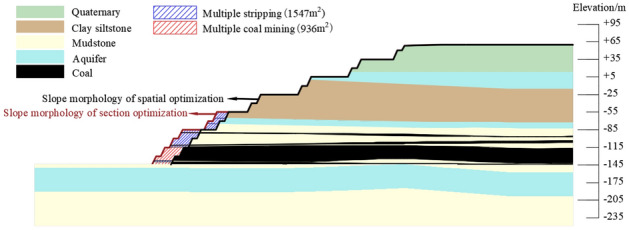
Comparison of the slope morphology and stripping amount between section optimized slope and spatial optimized slope.

The slope of the section morphology optimization was compared with the slope of the spatial morphology optimization. The stripping amount comparison was shown in Fig. 16. The slopes stability all meet the requirement of safety reserve coefficient 1.3. Compared with the section morphology optimization, after spatial morphology optimization the slope angle was increased by 2°, the stability coefficient was increased by 0.005. After section morphology optimization the stripping amount of measurement was increased by 1547 m^2^, and the recovery amount of coal resources was increased by 936 m^2^. In summary, the spatial morphology optimization scheme was better than the section morphology optimization scheme, and the economic benefit was significant.

## Conclusion

Taking the open-pit coal mine coal mine in Block I of Thar coal field in Pakistan as the research background. Used the method of combining two-dimensional rigid body limit equilibrium and three-dimensional numerical simulation, based on the section shape effect and spatial shape effect, the slope shape was optimized the design, and the economic benefits of the optimized slope shape were analyzed. The conclusions were as follows:

1. Based on the section morphology effect mechanism of slope stability, a section morphology optimization scheme was proposed, and the slope section morphology was optimized and designed.
2. A three-dimensional numerical simulation method was used to analyze the three-dimensional spatial effect of slope stability and reveal the influence of slope spatial geometry on slope stability. The spatial morphology of the slope was optimized and the evolution mechanism of slope instability was revealed.
3. Based on the effect of section morphology, breaking through the design of the traditional linear slope design of equal-split flat plate. The coal seam roof was used as a phased node, the slopes were staged into outer convex slope design. The examples showed that the slope angle of the optimized slope was 1° higher than that of the slope of preliminary design scheme, the stability coefficient was increased by 0.136, the stripping amount of section measurement was increased by 188 m^2^, and the recovery amount of coal resources was increased by 332 m^2^. The slope angle of the convex slope optimized by the section shape was 3° higher than that of the straight slope with equal flat plate, and the stability coefficient was equal. And the stripping amount of section measurement was increased by 2235 m^2^, and the recovery amount of coal resources was increased by 1790 m^2^. It highlights the potential economic benefits of section morphology effect.
4. Based on the effect of spatial morphology, the numerical simulation analysis showed that the slope stability coefficient gradually increased with the decrease of the tracking distance between the working slope and the inner dump, and decreased linearly with the increase of the slope angle. And the deformation and deformation range of deep rock mass decrease gradually. When the tracking distance was less than 100m, the three-dimensional spatial effect was remarkable. The examples showed that the slope angle of the slope after spatial morphology optimization was 3° higher than that of the slope of preliminary design scheme, the stability coefficient was increased by 0.141, the stripping amount of measurement was increased by 415 m^2^, and the recovery amount of coal resources was increased by 1188 m^2^. Compared with the straight slope with equal flat plate, after spatial morphology optimization the slope angle was increased by 5°, the stability coefficient of slope was increased by 0.005, the stripping amount was increased by 3636 m^2^, and the recovery amount of coal resources was increased by 2726 m^2^. Compared with the slope designed on the basis of section shape optimization, the slope angle of spatial shape optimization design was increased by 2°. The stability coefficient was increased by 0.005, the stripping amount was increased by 1547 m^2^, and the recovery amount of coal resources was increased by 936 m^2^. It highlights the potential economic benefits of spatial morphology effect.
5. Slope stability and landslide mode were mainly controlled by weak layers. The potential landslide mode was cutting-bedding sliding with C1 coal roof as the bottom interface. The calculation results of three-dimensional numerical simulation were closed to those of two-dimensional limit equilibrium method, and the position and morphology of the sliding surface were also close.

## Data Availability

The data used to support the findings of this study are available from the corresponding author upon request.
